# Ethnobotany of wild plants used for starting fermented beverages in Shui communities of southwest China

**DOI:** 10.1186/s13002-015-0028-0

**Published:** 2015-05-28

**Authors:** Liya Hong, Jingxian Zhuo, Qiyi Lei, Jiangju Zhou, Selena Ahmed, Chaoying Wang, Yuxiao Long, Feifei Li, Chunlin Long

**Affiliations:** College of Life and Environmental Sciences, Minzu University of China, Beijing, 100081 People’s Republic of China; Kunming Institute of Botany, Chinese Academy of Sciences, Kunming, 650201 People’s Republic of China; School of Agronomy and Biotechnology, Yunnan Agricultural University, Kunming, 650201 People’s Republic of China; School of Environment & Life Science, Kaili University, Guizhou, 556011 People’s Republic of China; Department of Health & Human Development, Montana State University, Bozeman, MT 59717 USA; School of Chemistry and Life Science, Guizhou Normal College, Guiyang, 550018 People’s Republic of China; Guizhou Institute for Advanced Study in Anthropology & Ethnology, Guizhou Normal College, Guiyang, 550018 People’s Republic of China

**Keywords:** Fermented beverages, Biodiversity, *Xiaoqu starter*, Traditional ethnobotanical knowledge, Cultural preservation

## Abstract

**Background:**

Shui communities of southwest China have an extensive history of using wild plants as starters (*Xiaoqu*) to prepare fermented beverages that serve important roles in interpersonal relationships and cultural events. While the practice of using wild plants as starters for the preparation of fermented beverages was once prevalent throughout China, this tradition has seen a decline nationally since the 1930s. The traditional technique of preparing fermented beverages from wild plant starters remains well preserved in the Shui communities in southwest China and provides insight on local human-environment interactions and conservation of plant biodiversity for cultural purposes. The present study sought to examine the ethnobotany of wild plants used as starters for the preparation of fermented beverages including an inventory of plants used as a starter in liquor fermentation and associated knowledge and practices.

**Methods:**

Field surveys were carried out that consisted of semi-structured surveys and plant species inventories. One hundred forty-nine informants in twenty Shui villages were interviewed between July 2012 and October 2014 to document knowledge associated with wild plants used as a liquor fermentation starter. The inventories involved plant voucher specimens and taxonomic identification of plant collections.

**Results:**

A total of 103 species in 57 botanical families of wild plants were inventoried and documented that are traditionally used as starters for preparing fermented beverages by Shui communities. The majority of the species (93.2%) have multiple uses in addition to being used as a starter with medicinal purposes being the most prevalent. Shui women are the major harvesters and users of wild plants used as starters for preparing fermented beverages and transfer knowledge orally from mother to daughter.

**Conclusions:**

Findings from this study can serve as a basis for future investigation on fermented beverages and foods and associated knowledge and cultural practices. However, with rapid development, utilization of wild plants and the cultural systems that support them are at risk of erosion. Cultural preservation practices are necessary in Shui communities for the continued use and transmission of this ethnobiological knowledge as well as associated biodiversity.

## Background

Fermented food and beverages that preserve diverse, locally available resources have been consumed for centuries worldwide as notable dietary components to support household food security and overall wellbeing [1–10]. Traditionally, such products were associated with cultural identity and social aspects of communities and were most often prepared at the household-scale through the action of microorganisms and their enzymes [10,11]. Key characteristics of fermented foods and beverages are enhancements to flavor and/or appearance, preserved quality, prolonged shelf-life, reduced cooking time and prebiotic and probiotic properties that have benefits for increasing digestability and bioavailablilty of certain nutrients [12–19]. Various cultures around the world prepare and consume fermented products to enhance their basic diet including as a side dish, condiment, pickle, confection and beverage. Knowledge on the preparation and attributes of fermented foods and beverages has been transferred from generation to generation and represents traditional ethnobiological knowledge.

In many minority socio-linguistic groups throughout China, the preparation and consumption of fermented alcoholic beverages are important cultural practices that define many social interactions including rituals during courtship, engagements, weddings, hospitality, funerals, ancestor worship and other ceremonies [20]. Various socio-linguistic groups have their own characteristics of preparing and consuming fermented alcoholic beverages that contribute to cultural identity such as Mongolian koumiss, Yi’s spicy liquor, Tibetan highland barley wine and Shui’s *Jiuqianjiu* liquor.

The Shui prepare fermented alcoholic beverages known in Chinese as *Jiuqianjiu* liquor and in Shui as *Kaojiuqian* that is made from water, rice and a special starter made of wild plants known as *Xiaoqu.* Although the origin is not clear, *Jiuqianjiu* liquor is a traditional fermented beverage that has long been prepared and consumed in Shui communities as an integral part of daily life as well as for celebratory reasons including the main Shui holiday that occurs in the lunar calendar during September. The production of *Jiuqianjiu* liquor involves harvesting wild plants and the wild type microbe inoculation of molds, yeasts and bacteria in a rice (or other grain) substrate. The whole process of making this starter from plant collecting to material mixing, shaping, ripening, drying and storing usually takes 3 months. The *Xiaoqu* starter is often regarded as the most important ingredient for determining the quality of the final beverage. The production of *Jiuqianjiu* liquor can be compared to the Japanese process of Koji. Diverse wild plants are used as a starter for preparing fermented alcoholic beverages in Shui and other socio-linguistic groups of southwestern China. Indigenous communities in southwestern China believe that the synergy of different wild plants with microorganisms modifies the environment for the microbes by providing nutrition and inhibiting the growth of detrimental microbes and ultimately can modify the flavor and health attributes of the final fermented product.

The use of wild plants as a starter for fermented beverages has a long history in China. The earliest known recording is by *Qi Min Yao Shu* during the years 533–544. With technological development, pure breeding fermentation technologies that were introduced to China in the 1930s replaced the use of wild plants as a fermentation starter in most areas throughout the country. In the present era, socio-economic and political influences in China’s rural areas are threatening traditional practices of preparing fermented foods and beverages along with their associated knowledge base.

Understanding the ethnobotany of wild plants used as a starter for preparing fermented beverages can serve as a basis for future studies and applications regarding fermented products. However, this information has not been documented in most communities where this practice remains prevalent. The present study tries to address this knowledge gap through an ethnobotanical investigation of wild plants used as starters for fermented beverages in indigenous Shui communities of southwest China. Specifically, our study aimed to address the following objectives in Shui communities: (1) characterize the plants used as starters to prepare fermented alcoholic beverages, (2) document associated ethnobotanical knowledge, and (3) record the processing of fermented alcoholic beverages.

## Materials and methods

### Study area and Shui people

Surveys were conducted in Sandu Shui Autonomous County located in the south of Guizhou Province of China (25°30′-25°10′ N, 107°40′-108°14′ E). It is in Qiannan Prefecture, Guizhou province where the Shui population is most densely settled and where the Shui people regard it as their cultural and linguistic center [21]. This area was selected for research because it is floristically rich with a cultural practice of drawing on this biodiversity for preparation of fermented beverages. Sandu Shui Autonomous County is characterized by numerous high mountains and large and small rivers. The climate is considered as a subtropical humid monsoon type with long summers and short winters. The complexity of the terrain, topography and altitude of the county has resulted in enormous variations of the climate. The country has a total area of 2,380 square kilometers with a total of 237,588 people living in 270 villages.

With an approximate population of 406,900, the Shui are one of the 55 officially recognized minority nationalities in China. Sandu Shui Autonomous County is the only county in China dominated by Shui people. The Shui people account for 65.93% of the total population of this prefecture; the remaining population consists of Han, Buyi, Miao, Yao and nine other minority socio-linguistic groups. They are distributed in Guizhou, Guangxi, Yunnan and Sichuan Provinces. The Shui language belongs to the Kam-Shui language grouping within the Sino-Tibetan language family [21, 22]. Due to the long communication with the Han, almost all Shui now know both Shui and Chinese Mandarin languages. While the Shui people have their own written language as found in the “Shui Book”, Chinese has become the written language in their daily life. The Shui also have their own calendar that dictates their ceremonies.

These Shui villages in mountainous valleys and basins are usually located near rivers and even today display the stilted wooden house style. The Shui at the study sites live in clusters of small-scaled villages. Until some decades ago there was a custom that a Shui woman should get married to one of her father’s sister’s son (cross-cousin marriage). Some have suggested that cross-cousin bonds reflected an original clan-based social organization with compact communities and villages typically of a few hundred related by bloodlines [21, 23]. Historically, they were not allowed to intermarry with other nationalities. Similar to other indigenous groups in the area, the Shui follow polytheism and animism with worship of ancestors and natural objects including mountains, rocks and ancient trees. Traditional lifestyle is still common in the area and the Shui people are fond of pickles and sour soup in their daily diet. Staple food of the Shuis is rice, together with different local vegetables and meat as protein source. Traditional practices are still common in Shui communities including the production of fermented alcoholic beverages.

**Fig. 1:**
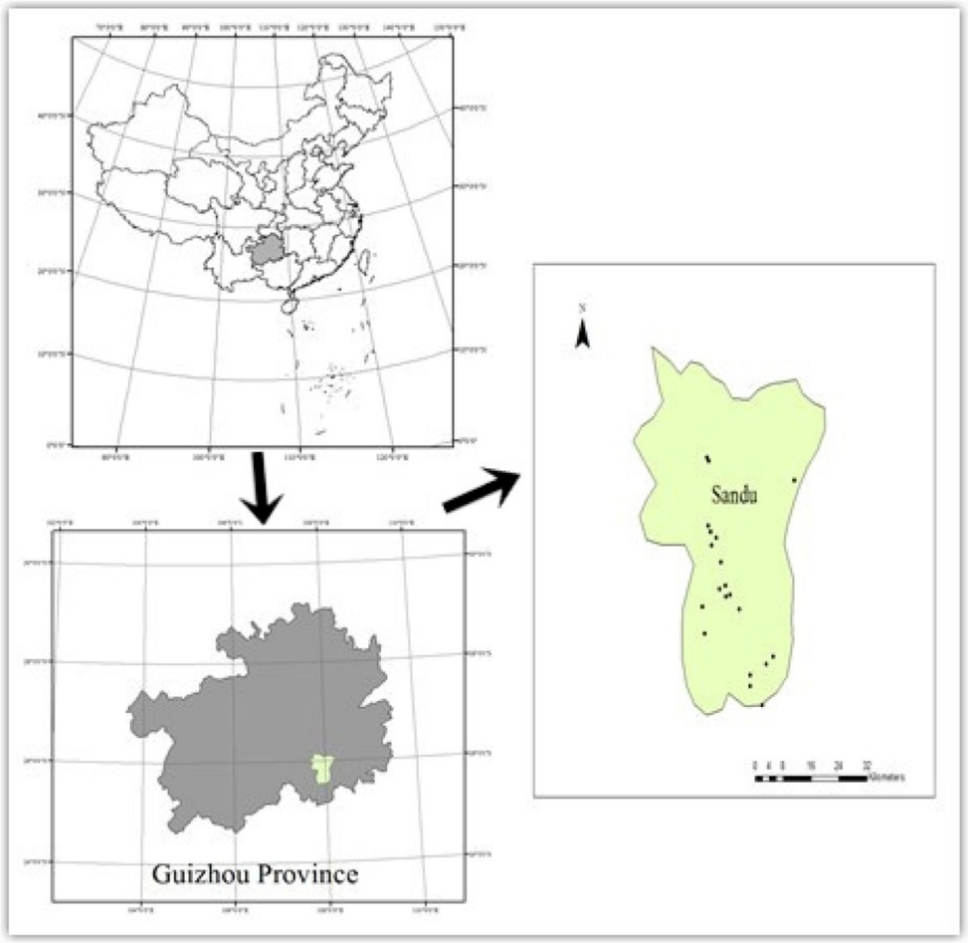
Sketch map of the study area, Sandu County of Guizhou

Field studies were carried out in 20 villages located in five townships in Sandu County including: Banlong, Bangao (Zhouqin town), Shuitiao, Dabian, Layou, Duzhai, Malian, Shuilong, Sanhe, Bamao, Banmiao, Miaoliang, Zenlei, Shuige, Bangao (Jiuqian town), Shuigen, Shuixi, Shuimei, Yanpai and Banqi villages. The villages were randomly selected from all Shui villages in Sandu County to carry out ethnobotanical investigations (Fig. 1). The majority of individuals in the villages are Shui. The population varies from 253 to 2,252 in each village. No notable geographical and environmental differences exist in the study area.

### Field surveys

**Fig. 2:**
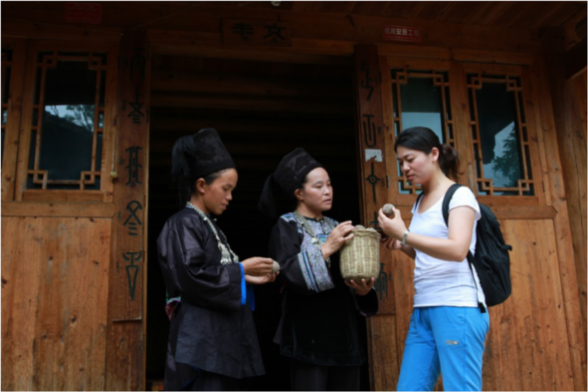
Key informant interview

Field surveys were carried out between July 2012 and October 2014 using participatory rural appraisal (PRA) and ethnobotanical methods [24–27] in order to document the species, habitats and varied uses of plants used as starters for preparing fermented beverages (Fig. 2). A total of 149 informants including 32 males and 117 females were interviewed (seven to ten people per village). Informants were between the ages of 23 and 84 years old. Fifty-three key informants were identified who were highly respected in their communities for their rich knowledge of plants used for starters for fermented beverages. Key informants included 38 village elders, 10 traditional brewers of fermented beverages and 5 managers of local liquor distilleries. In addition, ninety-six randomly selected households were surveyed. Permissions were provided by all participants in this study, including the Shui people.

**Fig. 3:**
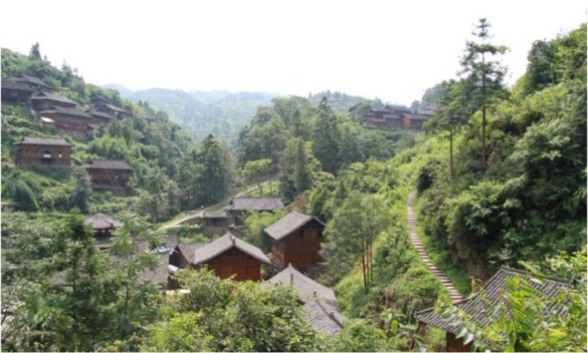
A Shui village investigated

During each visit, documented plant species were collected from different habitats around the study sites (Fig. 3). In addition, surveys documented vascular names, parts used, frequency of use and other values held by informants during interviews as well as through participant observation. Scientific name, botanical family name, growth forms, plant conservation status and other values, such as medicinal, edible, ornamental, spice, dyeing, herbal tea, fence and timber, were recorded for each plant species in Table 1. The collected ethnobotanical data were summarized using descriptive statistical analysis including frequency and their values. Frequency was used to determine the relative importance of plant species. Voucher specimens were collected, identified and deposited in the Herbarium at the College of Life and Environmental Sciences of Minzu University of China. Family assignation in this paper followed the *Flora of China* and TROPICOS.

**Table 1:** Plants used for liquor fermentation starter in Sandu County of Guizhou

No.	Chinese name		Shui name	Scientific name	Family name	Life form	Parts used	Frequency	Other value
1	Tuniuxi	土牛膝	ma^24^dian^43^	*Achyranthes aspera* L.	Amaranthaceae	Herb	Leaf	***	Clearing away heat and toxic materials; Diuretics; Cold; Fever; Amygdalitis; Mumps
2	Zhonghua-mihoutao	中华猕猴桃	faŋ^43^ma^24^	*Actinidia chinensis* Planch.	Actinidiaceae	Shrub	Leaf	****	The fruit is edible
3	Geyemihoutao	革叶猕猴桃	faŋ^43^ma^24^	*Actinidia rubricaulis* var. *coriacea* (Finet & Gagnep.) C.F. Liang	Actinidiaceae	Shrub	Leaf, Fruit	***	The fruit is edible
4	Longyacao	龙芽草	ɡa^31^jun^35^ɡaŋ^42^	*Agrimonia pilosa* Ledeb.	Rosaceae	Herb	Aerial part	*****	Stopping bleed
5	Xingxiang-tu’erfeng	杏香兔儿风	ʨyŋ^35^laŋ^31^thu^31^nu^24^luŋ^42^	*Ainsliaea fragrans* Champ. ex Benth.	Asteraceae	Herb	Aerial part	*****	Heat-clearing and detoxifying effect; Clearing away heat and toxic materials; Diuretics; Hematemesis; Traumatic injury
6	Sanyemutong	三叶木通	haŋ^31^wa^24^mei^33^thuŋ^55^	*Akebia trifoliata* (Thunb.) Koidz.	Lardizabalaceae	Liana	Leaf	***	Diuretics; Promoting lactation; Loosing bones and muscles; Removing dampness; Arthralgia
7	Ersexiangqing	二色香青	ja^55^sa^43^laŋ^35^ʨhu^53^	*Anaphalis bicolor* (Franch.) Diels	Asteraceae	Shrub	Whole plant	**	Removing dampness; Relieving summer-heat; Cough
8	Jinxiancao	金线草	niaŋ^55^ha^24^ɡaŋ^43^	*Antenoron filiforme* (Thunb.) Roberty & Vautier	Polygonaceae	Herb	Aerial part	****	Dispelling wind and cold; Removing dampness; Relieving pain; Stopping bleed; Eliminating stasis to stop pain
9	Zijinniu	紫金牛	ʨin^24^niaŋ^24^kui^42^	*Ardisia japonica* (Thunb.) Bl.	Myrsinaceae	Shrub	Aerial part	*****	Phthisis; Hemoptysis; Cough; Tracheitis; Removing dampness; Leukorrhea; Amenorrhoea; Diuretics
10	Qihao	奇蒿	ȵi^35^ai^35^	*Artemisia anomala* S. Moore	Asteraceae	Herb	Aerial part	***	Removing dampness; Promoting blood circulations; Eliminating stasis to stop pain; Irregular menstruation; Relieving pain
11	Hualianxixin	花脸细辛	nu^55^na^55^tə^35^na^24^	*Asarum splendens* (F. Maekawa) C. Y. Cheng & C. S. Yang	Aristolochiaceae	Herb	Whole plant	****	Dispelling wind and cold; Relieving pain; Warming lung for dispelling cold
12	Wulingxixin	五岭细辛	wə^33^nu^33^ tə^35^na^24^	*Asarum wulingense* C.F. Liang	Aristolochiaceae	Herb	Whole plant	***	Dispelling wind and cold; Relieving pain; Warming lung for dispelling cold
13	Tiejiaojue	铁角蕨	ɕie^24^pau^43^tə^35^	*Asplenium trichomanes* L.	Aspleniaceae	Herb	Whole plant	**	Heat-clearing and detoxifying effect; Removing dampness; Stopping bleed; Eliminating stasis to stop pain; Dysentery; Leukorrhea; Irregular menstruation; Backache
14	Ziwan	紫菀	ʨie^35^yue^55^	*Aster ageratoides* Turcz.	Asteraceae	Herb	Aerial part	****	Cold; Cough; Asthma; Consumptive disease; Blood vomitting
15	Sanmaiziwan	三脉紫菀	haŋ^43^mai^35^ ʨie^35^jue^55^	*Aster tataricus* L. f.	Asteraceae	Herb	Aerial part	***	Cold; Cough; Asthma; Consumptive disease; Blood vomitting
16	Xianghua	香桦	laŋ^24^huaˀ^55^	*Betula insignis* Franch.	Betulaceae	Tree	Leaf	*****	Cough; Asthma; Eliminating phlegm
17	Baiji	白及	ja^43^pau^43^	*Bletilla striata* (Thunb.) Rchb. f.	Orchidaceae	Herb	Root	*****	Stopping bleed; Anti-swelling agent; Promoting tissue regeneration
18	Laijiangteng	来江藤	daŋ^42^kui^33^jau^43^	*Brandisia hancei* Hook. f.	Scrophulariaceae	Shrub	Stem, Leaf	*****	Clearing away heat and toxic materials; Bone fracture; Hepatitis
19	Jinqianbao	金钱豹	niaŋ^24^ɕie^33^me^43^	*Campanumoea javanica* Bl.	Campanulaceae	Liana	Aerial part	***	Warming spleen and stomach for dispelling cold; Moistening lung to arrest cough; Inducing saliva and slakes thirst; Spleen tonic
20	Yanguantoucao	烟管头草	je^31^tau^24^ku^33^kaŋ^43^	*Carpesium cernuum* L.	Asteraceae	Herb	Aerial part	****	Clearing away heat and toxic materials; Anti-swelling agent; Anti-inflammation; Relieving pain; Cold; Fever; Toothache; Dysentery; Diuretics
21	Diaodenghua	吊灯花	tiau^35^phau^33^nuˀ^55^	*Ceropegia trichantha* Hemsl.	Asclepiadaceae	Liana	Whole plant	**	Clearing away heat and toxic materials; Bone fracture; Hepatitis; Ornamental
22	Rougui	肉桂	nai^55^kui^55^	*Cinnamomum cassia* (L.) D. Don	Lauraceae	Tree	Bark	*****	Relieving pain; Waist and knee pain; Stomachache; Dyspepsia; Amenorrhea; Spice
23	Ganju	柑橘	kaːŋ^55^	*Citrus reticulata* Blanco	Rutaceae	Tree	Leaf	*****	Regulating qi-flowing for eliminating phlegm; Promoting blood circulations; Anti-swelling agent; The fruit is edible
24	Dangshen	党参	taŋ^24^ʂen^24^	*Codonopsis pilosula* (Franch.) Nannf.	Campanulaceae	Liana	Aerial part	*****	Invigorating the blood; Spleen and stomach tonic; Weakness; The root is cooking for edible
25	Fanbaocao	饭包草	ma^33^kai^43^kei^33^	*Commelina bengalensis* L.	Commelinaceae	Herb	Aerial part	***	Clearing away heat and toxic materials; Diuretics; Anti-swelling agent; The leaves and stems are cooking as vegetables
26	Xianggang-sizhaohua	香港四照花	ɕi^24^ʦau^43^nuˀ^55^	*Cornus hongkongensis* Hemsl.	Cornaceae	Tree	Leaf, Fruit	**	Ornamental, The fruit is edible
27	Shamu	杉木	mei^35^faːi^24^	*Cunninghamia lanceolata* (Lamb.) Hook.	Taxodiaceae	Tree	Young leaf	***	Construction
28	Qinggang	青冈	mei^35^khei^24^	*Cyclobalanopsis glauca* (Thunb.) Oerst.	Fagaceae	Tree	Fruits, Barks	****	Clearing away heat and toxic materials
29	Yuyancao	鱼眼草	mei^35^la^31^kaŋ^43^	*Dichrocephala integrifolia* (L. f.) Kuntze	Asteraceae	Herb	Leaf	**	Clearing away heat and toxic materials; Rremoving dampness
30	Yeshi	野柿	mei^35^	*Diospyros kaki* var. *silvestris* Makino	Ebenaceae	Tree	Leaf	***	The fruit is edible
31	Hutuizi	胡颓子	nuei^35^liuˀ^55^la^33^	*Elaeagnus pungens* Thunb.	Elaeagnaceae	Shrub	Leaf, Fruit	*****	Dispelling wind and cold; Removing dampness; Removing blood stasis; Stopping bleed; Traumatic injury
32	Xiangru	香薷	laŋ^35^ʐu^43^	*Elsholtzia ciliata* (Thunb.) Hyl.	Lamiaceae	Herb	Stem, Leaf	***	Relieving exterior and sweating; Removing dampness; Warming spleen and stomach for dispelling cold; Diuretics; Anti-swelling agent
33	Jianyerong	尖叶榕	ɕie^43^wa^24^ʐuŋ^24^	*Ficus henryi* Warb.	Moraceae	Tree	Leaf, Fruit	****	Cough; Toothache; Removing dampness; The fruit is edible
34	Bili	薜荔	ɕue^35^li^43^	*Ficus pumila* L.	Moraceae	Shrub	Leaf	*****	Clearing away heat and toxic materials; Expelling damp; Diuretics; The achene is washed for cooking bean jelly
35	Diguo	地果	laŋ^43^aŋ^35^	*Ficus tikoua* Bureau	Moraceae	Herb	Stem, Leaf, Fruit	***	Heat-clearing and detoxifying effect; Removing dampness; Promoting blood circulations; Clearing away heat and toxic materials; The fruit is edible
36	Qianjinba	千斤拔	ɕie^35^ʨie^43^jun^33^	*Flemingia prostrata* Roxb.	Fabaceae	Shrub	Leaf	****	Dispelling wind and cold; Removing dampness; Removing blood stasis; Clearing away heat and toxic materials
37	Zhizi	栀子	mei^35^lə^43^	*Gardenia jasminoides* J. Ellis	Rubiaceae	Shrub	Leaf, Fruit	*****	Heat-clearing and detoxifying effect; Purging intense heat; Herbal tea; Ornamental
38	Weiyebaizhu	尾叶白珠	hen^55^wa^24^pa^33^lei^43^	*Gaultheria griffithiana* Wight	Ericaceae	Shrub	Lef	***	Removing dampness; Ornamental
39	Baiguobaizhu	白果白珠	pa^33^lei^43^ pa^33^lei^43^	*Gaultheria leucocarpa* Bl.	Ericaceae	Shrub	Leaf	***	Removing dampness; Ornamental
40	Maodadingcao	毛大丁草	ȵi^55^mo^33^ɕie^43^	*Gerbera piloselloides* (L.) Cass.	Asteraceae	Herb	Whole plant	*****	Clearing internal heat; Anti-inflammatory effects; Cold; Fever; Postpartum dysphoria
41	Dadingcao	大丁草	mo^33^ɕie^43^	*Gerbera anandria* (L.) Sch.-Bip.	Asteraceae	Herb	Whole plant	****	Heat-clearing and detoxifying effect; Removing dampness; Anti-swelling agent; Bleeding
42	Lubianqing	路边青	ʨia^35^khun^43^jy^43^	*Geum aleppicum* Jacq.	Rosaceae	Herb	Aerial part	*****	Irregular menstruation; Dispelling wind and cold; Removing dampness; Relieving pain
43	Suanpanzi	算盘子	mei^24^la^33^li^43^	*Glochidion puberum* (L.) Hutch.	Euphorbiaceae	Shrub	Stem, Leaf, Fruit	*****	Heat-clearing and detoxifying effect; Removing dampness; Dispelling wind and cold; Loosing bones and muscles; Leukorrhea; Dysmenorrhea
44	Shanxiaoju	山小橘	nu^55^tə^33^ʨiu^55^	*Glycosmis pentaphyll*a (Retz.) DC.	Rutaceae	Tree	Leaf	****	Eliminating stasis to stop pain; Anti-swelling agent; The fruit is edible
45	Nuomituan	糯米团	nu^55^man^33^kaŋ^43^	*Gonostegia hirta* (Bl. ex Hassk.) Miq.	Urticaceae	Herb	Whole plant	*****	Spleen tonic; Digestion; Heat-clearing and detoxifying effect; Removing dampness; Anti-swelling agent
46	Xiao’erxiancao	小二仙草	tə^33^ka^33^ɕie^43^kaŋ^43^	*Haloragis micrantha* Thunb.	Haloragidaceae	Herb	Whole plant	***	Diuretics; Expelling damp; Clearing away heat and toxic materials; Antidysmenorrhea; Promoting blood circulations
47	Changchunteng	常春藤	ma^43^lian^35^man^33^	*Hedera nepalensis* K. Koch	Araliaceae	Shrub	Whole plant	*	Dispelling wind and cold; Removing dampness; Promoting blood circulations; Anti-swelling agent; Ornamental
48	Ercao	耳草	khan^43^kaŋ^43^	*Hedyotis auricularia* L.	Rubiaceae	Tree	Leaf	***	Heat-clearing and detoxifying effect; Removing dampness; Anti-swelling agent
49	Zhiju	枳椇	ɕiu^43^	*Hovenia acerba* Lindl.	Rhamnaceae	Tree	Leaf, Fruit	****	Promoting blood circulations; Eliminating stasis to stop pain; Clearing heat and expelling damp; Asthma; Ornamental; Fence
50	Kuanluanye-changbing-shanmahuang	宽卵叶长柄山蚂蝗	sɿ^33^mi^43^	*Hylodesmum podocarpum* subsp. *fallax* (Schindl.) H. Ohashi & R.R. Mill	Fabaceae	Herb	Leaf	***	Dispelling wind and cold; Loosing bones and muscles; Clearing away heat and toxic materials; Anti-swelling agent; Traumatic injury; Removing dampness; Backache
51	Jianyechangbing-shanmahuang	尖叶长柄山蚂蝗	sɿ^33^mi^43^	*Hylodesmum podocarpum* subsp. *oxyphyllum* (DC.) H. Ohashi & R.R. Mill	Fabaceae	Herb	Leaf	***	Dispelling wind and cold; Loosing bones and muscles; Clearing away heat and toxic materials; Anti-swelling agent; Traumatic injury; Removing dampness
52	Tianjihuang	田基黄	khui^33^wan^33^tin^43^	*Hypericum japonicum* Thunb.	Clusiaceae	Herb	Aerial part	****	Heat-clearing and detoxifying effect; Removing dampness; Promoting blood circulations; Anti-swelling agent
53	Yuanbaocao	元宝草	juan^24^pin^33^ kaŋ^43^	*H*y*pericum sampsonii* Hance	Clusiaceae	Herb	Whole plant	***	Clearing away heat and toxic materials; Restoring menstrual flow; Loosing bones and muscles; Stopping bleed; Fever; Dysentery; Irregular menstruation,Leukorrhea
54	Baimao	白茅	hai^33^pa^43^	*Imperata cylindrica* (L.) P. Beauv.	Poaceae	herb	Root, Stem	*****	Stopping bleed; Heat-clearing and detoxifying effect; Diuretics
55	Jianyemulan	尖叶木蓝	wa^24^ɕie^43^mei^35^ʨhu^43^	*Indigofera zollingeriana* Miq.	Fabaceae	Shrub	Young stem, Leaf	***	Clearing away heat and toxic materials; Removing blood stasis; Stopping bleed
56	Yang’erju	羊耳菊	ma^35^hai^55^	*Inula cappa* (Buch.-Ham. ex D. Don) DC.	Asteraceae	Shrub	Aerial part	****	Dispelling wind and cold; Anti-swelling agent; Relieving pain
57	Yuanwei	鸢尾	wa^24^liu^43^	*Iris tectorum* Maxim.	Iridaceae	Herb	Root, Stem	****	Promoting blood circulations; Removing blood stasis; Dispelling wind and cold; Removing dampness; Clearing away heat and toxic materials; Digestion; Ornamental
58	Nanwuweizi	南五味子	nai^43^wo^24^wei^24^la^43^	*Kadsura longipedunculata* Finet & Gagnep.	Schisandraceae	Liana	Whole plant	****	Astringent; Inducing saliva and slakes thirst; Notifying kidney and spleen; The fruit is edible
59	Huorongcao	火绒草	jy^33^mau^43^kaŋ^43^	*Leontopodium leontopodioides* (Willd. ) Beauv.	Asteraceae	Herb	Whole plant	***	Clearing away heat and toxic materials
60	Yimucao	益母草	mai^33^kaŋ^43^	*Leonurus japonicus* Houtt.	Lamiaceae	Herb	Young stem, Leaf	*****	Promoting blood circulations; Removing blood stasis; Diuretics; Anti-dysmenorrhea
61	Yebaihe	野百合	nu^33^pa^43^	*Lilium brownii* F.E. Brown ex Miell.	Liliaceae	Herb	Bulb	****	Moistening lung to arrest cough; Heat-clearing and detoxifying effect; Tranquilizing the mind; Diuretics; The bulb is edible
62	Shanjijiao	山鸡椒	ɕiu^43^	*Litsea cubeba* (Lour.) Pers.	Lauraceae	Shrub	Leaf	*****	Warming spleen and stomach for dispelling cold; Dispelling wind and cold; Anti-swelling agent; Spice
63	Liuyerendong	柳叶忍冬	ha^33^tiau^33^ʨi^33^tin^43^kə^33^	*Lonicera lanceolata* Wall.	Caprifoliaceae	Tree	Stem, Leaf, flower	****	None
64	Danzhuye	淡竹叶	wa^33^fan^55^	*Lophatherum gracile* Brongn.	Poaceae	Herb	Whole plant	*****	Reducing pathogenic fire; Fever; Diuretics; Herbal tea
65	Haijinsha	海金沙	miau^33^nu^33^ka^55^	*Lygodium japonicum* (Thunb.) Sw.	Lygodiaceae	Herb	Aerial part	*****	Clearing away heat and toxic materials; Removing dampness; Relieving pain Urinary tract infections; Hepatitis; Nephritis edema; Diarrhea
66	Xiaoguo-shidagonglao	小果十大功劳	laŋ^33^tə^33^suŋ^33^ta^33^kuŋ^33^lau^24^	*Mahonia bodinieri* Gagnep.	Berberidaceae	Shrub	Whole plant	*****	Clearing away heat and toxic materials; Anti-swelling agent; Antidiarrheic; Dysentery; Hepatitis; Ornamental
67	Diren	地菍	lai^33^ʐ^24^	*Melastoma dodecandrum* Lour.	Melastomaceae	Shrub	Aerial part	*****	Removing toxicity; The fruit is edible
68	Shiqizhu	石荠苎	tin^43^ʨi^33^han^24^	*Mosla scabra* (Thunb.) C.Y. Wu & H.W. Li	Lamiaceae	Herb	Aerial part	*****	Cold; Malaria; Constipation; Hemafecia; Bleeding; Traumatic injury
69	Yangmei	杨梅	hai^24^	*Myrica rubra* (Lour.) Sieb. & Zucc.	Myricaceae	Tree	Leaf, Fruit	*****	Inducing saliva and slakes thirst; Digestion; The fruit is edible
70	Shenjue	肾蕨	ȵi^33^ʨian^43^	*Nephrolepis biserrata* (Sw.) Schott	Davalliaceae	Herb	Whole plant	*****	Cold; Fever; Cough; Tuberculosis; Dysentery; Enteritis; The tuber is edible
71	Xiaohuaihua	小槐花	nu^33^mei^33^suŋ^43^	*Ohwia caudata* (Thunb.) H. Ohashi	Fabaceae	Shrub	Leaf	*****	Hepatoprotective
72	Yezhiwei-jinfenjue	野雉尾金粉蕨	ȵin^24^ja^33^to^24^	*Onychium japonicum* (Thunb.) Kze.	Pteridaceae	Herb	Aerial part	***	Clearing away heat and toxic materials; Removing dampness; Stopping bleed; The leaves are cooked as vegetables
73	Jishiteng	鸡矢藤	jau^33^kai^24^	*Paederia foetida* L.	Rubiaceae	Liana	Aerial part	*****	Dispelling wind and cold; Removing dampness; Digestion; Clearing away heat and toxic materials; Anti-swelling agent; Promoting blood circulations; Relieving pain
74	Jinxiangcao	锦香草	laŋ^24^kaŋ^43^	*Phyllagathis cavaleriei* (Levl. et Van.) Guillaum.	Melastomaceae	Shrub	Whole plant	*****	Reducing pathogenic fire; Tonic
75	Changmang-jinxiangcao	长芒锦香草	kai^24^laŋ^24^kaŋ^43^	*Phyllagathis longiradiosa* C. Chen	Melastomaceae	Shrub	Leaf	***	Clearing away heat and toxic materials
76	Shanju	山蒟	man^33^luŋ^24^	*Piper hancei* Maxim.	Piperaceae	Liana	Aerial part	*****	Dispelling wind and cold; Removing dampness; Activating collaterals
77	Pingcheqian	平车前	ma^43^ma^35^	*Plantago depressa* Willd.	Plantaginaceae	Herb	Aerial part	*****	Clearing away heat and toxic materials; Diuretics; The whole plants is cooked as vegetables
78	Jiegeng	桔梗	haŋ^33^tie^24^	*Platycodon grandiflorus* (Jacq.) A. DC.	Campanulaceae	Herb	Root	*****	Cough;Eliminating phlegm; Ornamental
79	Guazijin	瓜子金	ha^33^ɣə^33^ti^33^	*Polygala japonica* Houtt.	Polygalaceae	Herb	Aerial part	*****	Promoting blood circulations; Eliminating stasis to stop pain; Eliminating phlegm and relieve cough; Clearing away heat and toxic materials; Relieving pain
80	Laliao	辣蓼	lie^24^liauˀ^55^	*Polygonum hydropiper* L.	Polygonaceae	Herb	Aerial part	*****	Clearing away heat and toxic materials; Eliminating stasis to stop pain;stopping bleed; Dysentery; Traumatic injury; Spice
81	Machixian	马齿苋	ma^33^jun^33^ȵie^24^	*Portulaca oleracea* L.	Portulacaceae	Herb	Aerial part	*****	Clearing away heat and toxic materials; Promoting circulation and removing stasis; Anti-swelling agent; The aerial part is cooked as vegetabl
82	Shiganzi	石柑子	tin^33^kai^33^la^55^	*Pothos chinensis* (Raf.) Merr.	Araceae	Liana	Stems, Leaf	****	Dispelling wind and cold; Removing dampness; Promoting blood circulations; Eliminating stasis to stop pain; Digestion; Cough
83	Ge	葛	pei^33^hai^55^	*Pueraria montana* (Lour.) Merr.	Fabaceae	Liana	Leaf	*****	Fever; Inducing saliva and slakes thirst; Inducing saliva and slakes thirst; Antidiarrheic; Kudzu powder is edible
84	Wannianqing	万年青	ɕin^33^pe^31^ʨhiu^55^	*Rohdea japonica* (Thunb.) Roth	Liliaceae	Herb	Leaf	****	Clearing away heat and toxic materials; Eliminating stasis to stop pain; Relieving pain
85	Cili	刺梨	nei^24^	*Rosa roxburghii* Tratt.	Rosaceae	Shrub	Fruit	*****	Heat-clearing and detoxifying effect; Inducing saliva and slakes thirst; Digestion; The fruit is edible
86	Jinyingzi	金樱子	ȵy^33^məŋ^33^ja^43^	*Rosa laevigata* Michx.	Rosaceae	Shrub	Leaf, Fruit	*****	Promoting blood circulations; Eliminating stasis to stop pain; Dispelling wind and cold; Removing dampness; Clearing away heat and toxic; The fruit is ediblematerials
87	Cuyexuangouzi	粗叶悬钩子	luei^31^wa^24^lauˀ^55^	*Rubus alceaefolius* Poir.	Rosaceae	Shrub	Root, Leaf	***	Promoting blood circulations; Removing blood stasis; Heat-clearing and detoxifying effect; Stopping bleed; The fruit is edible
88	Shanmei	山莓	tuŋ^33^kaˀ^55^	*Rubus corchorifolius* L. f.	Rosaceae	Shrub	Stem, Leaf, Fruit	****	Promoting blood circulations; Stopping bleed; Dispelling wind and cold; Removing dampness; The fruit is edible
89	Tuoyuan-xuangouzi	椭圆悬钩子	lun^33^laŋ^24^ku^33^lu^33^	*Rubus ellipticus* Sm.	Rosaceae	Shrub	Young stem, Leaf	***	Anti-swelling agent; Relieving pain; Antidiarrheic; The fruit is edible
90	Huangguo-xuangouzi	黄果悬钩子	lun^33^laŋ^24^man^33^	*Rubus xanthocarpus* Bureau & Franch.	Rosaceae	Shrub	Leaf	***	Anti-inflammatory effects; Relieving pain; The fruit is edible
91	Hongpaociteng	红泡刺藤	jau^43^lun^33^han^33^	*Rubus niveus* Thunb.	Rosaceae	Shrub	Leaf, Fruit	****	Dispelling wind and cold; Removing dampness; Clearing away heat and toxic materials; Dysentery; The fruit is edible
92	Daxueteng	大血藤	ma^31^len^24^ʨieˀ^55^	*Sargentodoxa cuneata* (Oliv.) Rehder & E.H. Wilson	Lardizabalaceae	Liana	Stem, Leaf	****	Clearing away heat and toxic materials; Promoting blood circulations; Dredging collaterals; Dispelling wind and cold; Convulsive disease
93	Xiaoxueteng	小血藤	ma^31^jiu^43^ ʨieˀ^55^	*Schisandra propinqua* (Wall.) Baillon	Schisandraceae	Liana	Aerial part	****	Clearing away heat and toxic materials; Anti-swelling agent; Eliminating stasis to stop pain; Stopping bleed
94	Tiegusan	铁箍散	ɕin^33^nuei^33^ku^33^san^33^	*Schisandra propinqua* subsp. *sinensis* (Oliv.) R.M.K. Saunders	Schisandraceae	Liana	Stem, Leaf, Fruit	****	Dispelling wind and cold; Promoting blood circulations; Clearing away heat and toxic materials; Anti-swelling agent; Relieving pain; Irregular menstruation
95	Sanmaibaqia	三脉菝葜	fa^24^mə^33^en^24^	*Smilax trinervula* Miqual	Liliaceae	Shrub	Leaf	****	None
96	Citianqie	刺天茄	suŋ^31^wən^24^ʨia^24^	*Solanum violaceum* Ortega	Solanaceae	Shrub	Leaf	***	Anti-inflammatory effects; Clearing away heat and toxic materials; Relieving pain
97	Baitan	白檀	pa^33^than^24^	*Symplocos paniculata* Miq.	Symplocaceae	Shrub	Leaf	*****	Clearing away heat and toxic materials; Removing blood stasis; Dispelling wind and cold
98	Hongdoushan	红豆杉	mei^24^nu^33^	*Taxus wallichiana* Zucc. var. *chinensis* (Pilg.) Florin	Taxaceae	Tree	Young leaf	*****	Digestion; Ascariasis; Ornamental
99	Qinglichai	青篱柴	ʨhiu^24^li^33^lin^24^	*Tirpitzia sinensis* (Hemsl.) Hallier f.	Linaceae	Shrub	Stem, leaf	***	Anti-swelling agent; Relieving pain; Bone fracture; Ornamental
100	Xiangchun	香椿	mei^24^ȵiu^43^	*Toona sinensis* (A. Juss.) M. Roem.	Meliaceae	Tree	Leaf	*****	Cold; Removing dampness; Stomachache; Dysentery; The leaves is cooked as vegetable
101	Mabiancao	马鞭草	ma^24^pian^33^kaŋ^43^	*Verbena officinalis* L.	Verbenaceae	Herb	Aerial part	*****	Promoting blood circulations; Eliminating stasis to stop pain; Malaria; Clearing away heat and toxic materials; Diuretics; Anti-swelling agent
102	Lanshu	蓝树	mei^24^ʨhiu^24^	*Wrightia laevis* Hook. f.	Apocynaceae	Tree	Leaf	*****	Traumatic injury; Stopping bleed; Dye plant
103	Huajiao	花椒	ɕiu^24^	*Zanthoxylum bungeanum* Maxim.	Rutaceae	Tree	Leaf	*****	Digestion; Relieving pain; Insecticidal; Anti-itch; Spice

Frequency: ***** > 75% of respondents; **** > 50% of respondents; *** > 25% of respondents; ** > 12.5% of respondents; * < 12.5% of respondents, but at least 5 respondents

(Ranked by scientific names alphabetically, followed by generic and species names)

## Results and discussion

### Diversity of plants used as starters for preparing fermented beverages

**Fig. 4:**
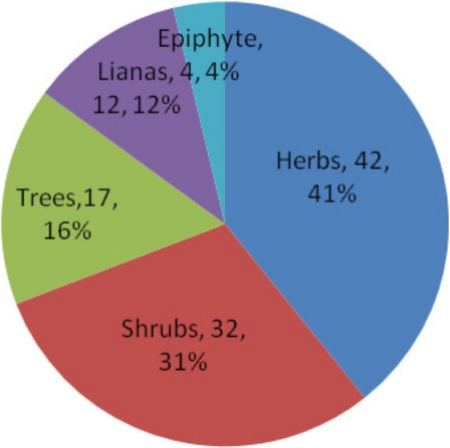
Life forms of wild plants used for liquor fermentation starter

A total of 103 wild-harvested plant species were documented as starters for preparing fermented alcoholic beverages at the study sites. This includes species distributed in 88 genera and 57 families consisting of 97 species of angiosperms, 2 species of gymnosperms, and 4 species of pteridophytes. The majority of plants belonged to the Asteraceae (12 species), Rosaceae (9), Fabaceae (6), Melastomaceae (3), Moraceae (3) and Rutaceae (3). Table 1 lists the Chinese name, Shui name, scientific name, family name, habitat, plant parts used, frequency of utilization and their values. The recorded species occur as various life forms with the majority being herbaceous species (42 %) and the remaining occurring as shrubs (32 %), trees (17 %), lianas (12 %), and epiphytes (4 %) (Fig. 4). All of the total 103 plant species were wild harvested from montane forests, wetlands, shrub lands, and wastelands. Most of the wild plant species had wide distribution in the study sites and were easily accessible.

**Fig. 5:**
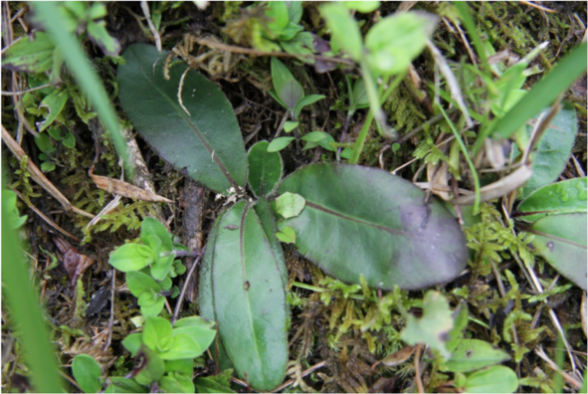
*Gerbera piloselloides*

**Fig. 6:**
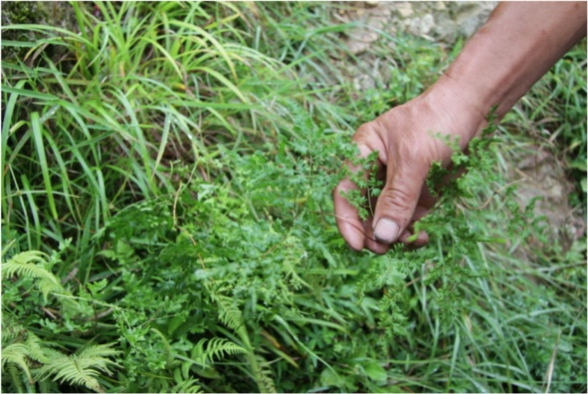
*Lygodium japonicum*

**Fig. 7:**
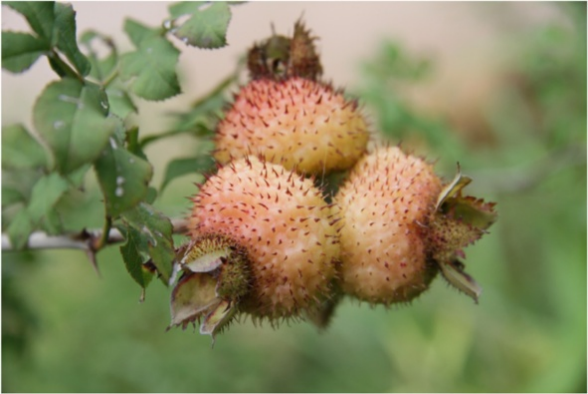
*Rosa roxburgii*

**Fig. 8:**
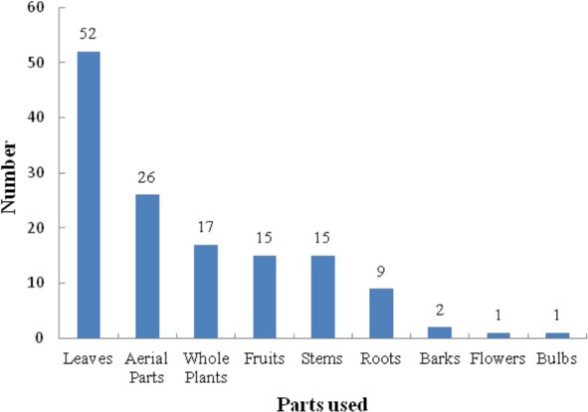
Plant parts used for liquor fermented starter

The average number of species mentioned per informant for use as a starter for the preparation of fermented alcoholic beverages was 15 while the average number for key informants was 54 species. The most frequently mentioned plants by informants include *Gerbera piloselloides* (Fig. 5)*, Lygodium japonicum* (Fig. 6)*, Rosa roxburghii* (Fig. 7)*, Paederia foetida, Zanthoxylum bungeanum*, *Plantago depressa* and *Platycodon grandiflorus*. Informants use varied plant parts for preparing the fermented culture including leaves, roots, barks and fruits (Fig. 8). The majority of plants are harvested for their leaves (52 species documented) followed by aerial parts (26). Informants also use whole plants (17), fruits (15) as well as other parts.

Documentation of local wild floristic diversity used by Shui communities as starters for the preparation of fermented beverages is important to support conservation and cultural revitalization efforts in the study site communities. All of the 103 plant species documented as starters are wild harvested and reflect the rich biological diversity in the study area. The greater use of herbaceous plants compared to other types of plants such as woody plant species might be due to their ease of collection, higher abundance, and high effectiveness in comparison to other life-forms. Previous ethnobotanical studies have found that weedy and herbaceous plants are often the majority of plants collected for food, medicine, and other purposes [27]. In addition, various plant parts are use for fermentation with the leaf being the most prevalent plant part used. Previous research in minority socio-linguistic communities in China has shown similar results of leaves being the most prevalent part harvested; this might be due to ease of collection and less threat presented to harvested resources as the practice of harvesting the whole plant or roots presents greater threats to survival [28–30]. In addition, the prevalence of collecting herbaceous plants versus other plant types might also be due to socio-cultural beliefs and practices of the Shui as well as environmental factors. Communities may harvest shrubs, trees, and lianas for their ability to withstand long dry seasons that result in their availability throughout the year as well as increased availability during the harvest period that wild plants are generally collected for starters. Seasons when plants bloomed and fruited, typically during the summer, are also when it is easiest to identify plants for collection. Furthermore, the summer is the best season for microbial fermentation of food and beverages because of the higher temperature promotes fermentation activity. Preparation during the summer also fits within the local Shui calendar as this is after the harvest of rice that rice is used as a raw material input for fermented beverages.

### Multiple uses and economic value of the wild plants

In addition to fermentation purposes, informants use the documented wild plants for multiple other purposes. Almost all documented wild plants (98.1 %) are locally valued for their medicinal, edible, ornamental, spice, dye, herbal tea, fence and timber uses with medicinal purposes for the prevention and treatment of different health conditions being the most prevalent use (Table 2). Specifically, the most common medicinal function of reported plants is to clear heat away followed by detoxification. Some plants had a single medicinal function while others had multiple medicinal functions. For example, *Lygodium japonicum* is used to treat urinary tract infections, hepatitis, nephritis edema, diarrhea, and other health conditions while *Melastoma dodecandrum* is used for removing toxicity. A few documented species are used for both food and medicine such as *Litsea cubeba*, *Imperata cylindrica*, *Kadsura longipedunculata*, *Cinnamomum cassia*, *Codonopsis pilosula*, *Ficus pumila* and *Rosa roxburghii*. In addition to food and medicinal purposes and for making starters, the documented species are valued for providing household income, celebrating local festivals and for construction material. For example, *Paederia foetida* is used for making festival rice cakes by the Shui and *Cunninghamia lanceolata* is the main timber tree species used for local construction. *Wrightia laevis* is used to dye the customary clothing of the Shui and results in a dark blue stain that is valued for clothing by Shui informants (Fig. 2).

**Table 2:** Types of multiple uses for wild plants in Sandu County of Guizhou

Value	Number	Percentage (%)
beverage starter	103	100.0
Medicinal	96	93.2
Edible	30	29.1
Ornamental	11	10.7
Spice	4	3.9
Construction	2	1.9
Herbal tea	2	1.9
Fence	1	1.0
Dyeing	1	1.0

Informants reported that fermented alcoholic beverages prepared from wild plants had enhanced taste that is smoother compared to beverages prepared without these plants. Most informants reported that using a greater number of plant species as a starter results in improved quality of the final fermented alcoholic beverage. In addition, informants reported that wild species are conveniently located near households in the study area and are available free of cost. Nowadays, informants travel several dozens of kilometers to procure these plants as not all plants used are located near their households and because of habitat destruction near their households. Compared to cultivated plants, wild plants require less management, are usually not directly impacted by agro-chemical pollution, and are a rich source of micronutrients [27]. Interviews and participant observation in local fields [31, 32] found that none of the documented plants were cultivated. In addition, participant observation in local markets [33] found that none of the documented plants were sold for liquor fermentation.

### Traditional harvest and preparation of *Jiuqianjiu*

Shui families are able to provide the daily quantity and quality of *Jiuqianjiu* liquor needed from locally available resources that are relatively cheap as well as with the use of simple equipment. Given the accessibility of needed plant material and affordability of equipment in making fermented alcoholic beverages, Shui women can support the social life of their families with this traditional practice. Preparation starts with harvesting wild plants. Harvested plants are usually dried in the sun after collection and are then powdered and stored in a cool dry place for preservation of flavor and health attributes.

The Shui have developed their traditional production sequence of liquor-making according to their farming seasons. Wild plants for starters are harvested between May to September in the study areas. Usually, local households make the beverage starter in summer and brew the liquor during the autumn. The Shui practice of preparing starters in the summer in congruent to previous studies that widely supported that microorganisms bred more quickly in summer and thereby shorten the production cycle and provided greater liquor yield [34, 35]. That is because the temperature in summer is believed to be better due to certain criteria, such as rapid fermentation.

Locals harvest wild plants according to local socially-negotiated protocols that prevent overharvesting of common resources including only harvesting mature individuals when whole plants are needed. In addition, locals prepare enough liquor fermentation starter at one time for three years to ensure plenty of time for plant growth. Local practices of regulating harvesting of common wild plant resources and making enough liquor starter at a time for three years to allow time for plant growth highlights the sustainability ethos of local communities. It is vital to conserve these wild plants while protecting their habitats. They must also be managed in sustainable ways to promote their use in suitable method [36]. It is suggested recruiting ethnobotanists and experts to train local communities on the sustainable utilization of wild plant resources to complement traditional practices [37, 38].

Both non-sticky rice and sticky rice are used by informants as the primary ingredient of *Jiuqianjiu* liquor with *Xiaoqu* being the starter agent during the fermentation process. Glutinous rice is the favored rice for liquor brewing by Shui informants because locals perceive it results in a higher quality product that has better taste. There are 15 glutinous rice varieties in Shui communities in Sandu County compared to 7 varieties in other areas of Sandu County. However, the lower yield of glutinous rice results in a relatively higher cost than common rice in the process of liquor making. These glutinous rice varieties are generally reserved for use in festival foods and desserts as well as serving as the main raw material for liquor making in the study area. Locals especially value black glutinous rice. Previous nutrition analysis and phytochemical investigation on black glutinous rice has relatively higher levels of polyphenols and anthocyanin content compared to other varieties of rice; these compounds are known for their medicinal functions in humans including antioxidant activity, reducing cholesterol levels and inhibiting cancer cell proliferation [39–44]. In addition, black glutinous rice has a number of nutritional advantages compared to many other rice varieties including higher content of protein, vitamins and minerals [45].

Polished rice is used for preparing *Jiuqianjiu* liquor that is thought to remove substances in rice aside from the starch that are regarded as undesirable substances for liquor brewing. Following polishing, the rice is washed and immerged using cold spring water at room temperature for 24 h. After steeping, excess water is drained off for 4 h before cooking the rice with steam for 1 h in a wood rice steamer. Subsequently, the steamed rice is spread out for cooling in the open air until the temperature falls to nearly 35 °C.

**Fig. 9:**
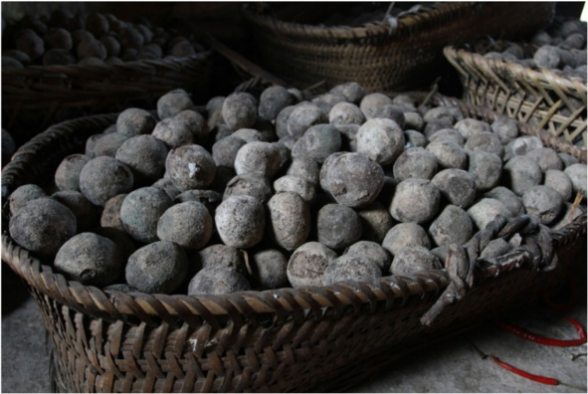
The liquor fermentation starter, or *Xiqoqu* starter

Beverage starters usually occur as a dried ball or cake of flour cultured with various molds, yeasts and bacteria (Fig. 9). *Xiaoqu*, which is the dominant starter for *Jiuqianjiu* liquor at the study sites, is prepared by a wild type microbe inoculation of molds, yeasts and bacteria as well as their growth on rice or other grains. The starter is crushed and added to inoculate the cereal substrate to initiate fermentation into liquor.

Informants reported a general method for mixing material for starters that includes boiling the plant mixture powder (5 %, w/w) with the spring water (48 %, w/w) for 3 h and then mixing the rice bran and glutinous rice flour (45 %, w/w) with the last year’s starter (2 %, w/w). The grain mix is then pulverized and the plant materials are stirred in. A wild type microbe inoculation method is used to promote the growth of molds, yeasts and other bacteria. The mixed material is typically milled and pressed into a mould of egg-size by hand. Then, it is incubated at 28-30  °C for 7 days in a room with special climatic conditions. After 7 days of incubation the mixture is dried at 45 °C until the humidity is lower than 15 % (w/w). The starter is then stored in a cold and dry place until use for liquor making within the next three years.

The steamed rice is mixed with 1 % (w/w) of the starter mixture that starts the processes of saccharification, acidification, and fermentation of the steamed rice. Then, the mixture is placed in wooden cask that is placed in a pit dug in the ground at 40 °C for 2 days. The saccharified mixture is then mixed with 120 % (w/w) cold spring water to form the thick slurry. Subsequently, the slurry is fermented at room temperature for 15 days in a semi-solid state. This slurry is carefully stirred by local liquor makers to aerate and maintain an optimal level of oxygen and carbon dioxide in the mixture, as well as to maintain an even temperature throughout the fermenting process. After fermentation, the rice wine mash is filtered using a bamboo basket for the purpose of removing the fermented grains. Then, a part of filtered liquid is evaporated to the dense liquid while the other is used for distilling the liquor. The two parts are then mixed at a certain proportion to increase the concentration of alcohol in the mash and halt the fermentation process.

**Fig. 10:**
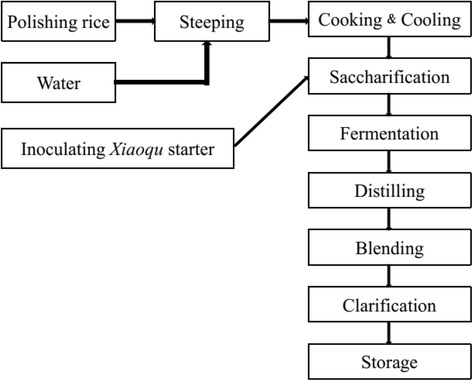
The processing procedures of *Jiuqianjiu* liquor in Sandu County of Guizhou

The process described above (Fig. 10) leaves a notable quantity of unfermented saccharides and chemical compounds from the plants and rice, thus producing a sweet taste and mouth-feel that is distinct from other forms of rice wine. Finally, the slightly turbid rice liquor is pumped through pottery casks for clarification and storage. Usually, the liquor is aged and stored underground for several years at a storage temperature of 13-18 °C. Unlike common rice wine, *Jiuqianjiu* liquor is distilled and mixed with the fermentation concentrate resulting from the starter. During storage, the liquor matures gradually and adopts a smoother taste. The various types of *Jiuqianjiu* liquor at the study sites vary in color from beige to yellowish-brown depending on the weight ratio of the distilled liquor versus the fermented concentrate. The final product is consumed at room temperature or after being warmed. Grains spent during fermentation are usually used as fodder for livestock at the study sites.

### Shui drinking culture and use of *Jiuqianjiu*

Preparation and consumption of *Jiuqianjiu* liquor is an important part of traditional social life and interpersonal relations at the study sites. There is a cultural practice to socially consume fermented alcoholic beverages in Shui communities that is known as *zhuan zhuan jiu*. This practice proceeds by all participants having a pot filled with local liquor on the right hand and feeding this beverage to their neighbor to the right. Informants reported that this practice reflects their respect to guests and is offered as a gesture of equality between the hosts and the guests.

Another use involving *Jiuqianjiu* liquor is by mothers after giving birth to improve their postpartum recovery. After the third day, mothers can go back to work outdoors if they drank *Jiuqianjiu* liquor after delivery. In comparison, mothers are commonly not allowed to work until 1 month after birth in China. All the informants indicated that traditional *Jiuqianjiu* liquor can make women during their puerperium (approximately 6 weeks after birth) and their babies healthy and strong. Informants believe that wild plants play a role in this function. This finding of the role of wild plants after birth is consistent with the findings of Yao people in China, whereas the difference is the mode of administration between Yao people and Shui people. Yao people use medicinal plants for medicine bath whereas the Shui consume these plants via fermented alcoholic beverages [38, 46, 47].

### Gender and knowledge transmission

Women are the primary harvesters of plants used as starters for making *Jiuqianjiu* liquor as well as the makers of these beverages at the study sites. While most of the male Shui informants carry out housework such as cooking, few male informants had knowledge of how to collect plants for starters and prepare fermented alcoholic beverages. Knowledge of plants used as starters is orally transferred from mother to daughter.

Findings show a significant correlation between informant age and plant knowledge. The distribution of informants in age, gender and education class is shown in Table 3. This study found that most informants who were known to have substantive knowledge on making of *Jiuqianjiu* liquor and fermented beverages as well as those that were practicing this tradition were primarily older than 50 years old. Female informants represented 78.5 % of the total sample group and the majority of key informants interviewed were females above 65 years old that had little to no formal education. Informants indicated that an increasing amount of young girls studied in school and did not learn the traditional practice of liquor making. This study further showed that female Shui informants who were well educated and aged between 20 and 35 knew how to brew *Jiuqianjiu* liquor but were not able to identify wild plants used as a beverage starter. Most young females at the study sites no longer learn about these plants and how to prepare traditional fermented beverages; rather, they purchase new commercial starters or fermented alcoholic beverages from the market that are increasingly available. Informants reported that this was mostly because of migration out of rural communities for jobs. Consequently, the traditional Shui ethnobotanical base is failing to be transferred. Knowledge of wild plants should thus be recorded and conveyed in Shui areas for their sustainable use and continuation of cultural practices linked to local biodiversity.

**Table 3:** Demographic profile of informants

Indicator	Description	Frequency (%)
Age	20-29	7(4.7)
	30-39	23(15.4)
	40-49	28(18.8)
	50-59	41(27.5)
	60-69	27(18.1)
	70-79	15(10.1)
	≥80	8(5.4)
Gender	Male	32(21.5)
	Female	117(78.5)
Education	None	31(20.8)
	Primary	85(57.0)
	Secondary	21(14.1)
	Tertiary	12(8.1)

### Scale of production

Shui informants prepare fermented beverages for household consumption and commercially in small-scale local distilleries. Small-scale liquor distilleries are distributed widely throughout the study sites and surrounding areas that provide local people with high-quality and relatively affordable local liquor. Wild plants are collected around residences of producers both for when they are preparing beverages for household use as well as for sale in the market. *Jiuqianjiu* liquor, like most other local alcoholic beverages in minority areas in China, is still produced with traditional equipment that is not backed by scientific knowledge of the process and has little regard for hygiene. Whereas small-scale manufacture has the advantages of short distribution lines, income generation for families, etc., urbanization and increased regional tourism and the resulting growing demand for ready-to-consume high-quality foods requires larger-scale controlled industrial production.

The final products are dependent on local climatic conditions and therefore the sensory characteristics and the quality are variable. The major problem associated with the traditional method of producing *Jiuqianjiu* liquor is that the product can never be uniform nor predicted because of the diversity of involved microorganisms and difficulties in controlling this population. Upgrading of traditional home-scale processes is needed so that it will lead to a better standardization and safety of a product that is already acceptable to the cultural tastes of local communities. In addition, this standardization process is helpful to continue to maintain and strengthen cultural heritage of Shui communities while providing a product that can compete successfully with industrial versions. However, *Jiuqianjiu* liquor is a homemade activity and uncontrolled microbial inoculation based on spontaneous fermentation. The ecosystem in *Jiuqianjiu* liquor represents a source of biodiversity that can be exploited to create such functional starter cultures. The microbiological details of this highly sophisticated fermentation technology has remained undocumented. In fact, *Jiuqianjiu* liquor has the potential to become an important source of new valuable microbial strains for biotechnology. Future studies are needed to evaluate the phytochemical profiles, bioactivity, stability and safety of fermented wild plants and their synergies.

Wild plants are threatened by various socio-ecological reasons including climate change, land use change, habitat destruction, overharvesting and etc. [48–52]. The construction of highways and other infrastructure as well as deforestation for agricultural purposes is severely threatening wild habitats for plants in Guizhou Province. In addition, unsustainable harvest of plant species with market value also contributes to a decrease of these wild resources. Both *ex situ* and *in situ* conservation methods are needed in the natural or farmed environment to preserve the biodiversity of wild plants used for preparing fermented beverages and associated cultural systems. It is necessary that local people support and participate in these conservation initiatives for the most successful results.

## Conclusion

This paper provides documentation of the diversity of wild plants used as starters to prepare fermented beverages by Shui communities of Sandu County in Southwest China. Findings highlight the rich biodiversity and habitats that local communities draw upon from their surroundings as part of their cultural life to support interpersonal communication and celebrate key occasions. Women’s role as the primary producers involved in making fermented beverages reflects gendered knowledge that is related to societal life and relations to kin. While knowledge of plants used for liquor making has traditionally been orally transferred from mother to daughter, this knowledge is threatened as the younger generations move away from rural areas in search of jobs and a different lifestyle, a pattern witnessed in rural communities worldwide. Efforts are needed to enhance the transmission of ethnobotancial knowledge in Shui communities towards conservation of biodiversity and associated preservation of cultural systems. Increased interest in natural products and artisanal beverages as well as increased regional tourism is attracting new interest in wild plants used in the processing of fermented foods and beverages. If developed with local community interests and conservation in mind, these commercialization and tourism efforts have the potential of helping preserve traditional ethnobotanical knowledge as well as associated biodiversity. Future studies are needed to evaluate the phytochemical profiles, bioactivity, stability and safety of fermented wild plants and their potential for other fermented foods and beverages as well as medicinal purposes. In addition, it is necessary to develop standards for large-scale production and commercialization of these non-timber forest products. These future studies would help provide guidelines for community-based production and ultimately preservation of biological and cultural diversity.

### Consent

Permissions were provided by all participants in this study, including the Shui people. Consent was obtained from the participants prior to this study being carried out. The authors have all copyrights.
